# “Too little, too late”: youth retrospectives on school attendance problems and professional support received

**DOI:** 10.3389/frcha.2025.1595289

**Published:** 2025-05-27

**Authors:** Selina Eckhoff Hamadi, Trude Havik, Solveig Holen

**Affiliations:** ^1^ Norwegian Centre for Learning Environment and Behavioral Research in Education, University of Stavanger, Stavanger, Norway; ^2^ Regional Centre for Child and Adolescent Mental Health, Eastern and Southern Norway, Oslo, Norway

**Keywords:** school attendance problems, school refusal, truancy, youth perspectives, anxiety, alternative provision

## Abstract

**Introduction:**

School attendance problems (SAPs) are a vexing issue that pose significant challenges for youth, families, and professional stakeholders. Despite growing research efforts, studies that explore the perspectives of youth on SAPs remain limited, particularly in Nordic countries. This qualitative study investigated Norwegian youths' retrospectives on the development and persistence of SAPs and the support they received from professionals in addressing their challenges.

**Methods:**

Semi-structured interviews were conducted with 10 youth (aged 12–22) with a history of SAPs in compulsory education. Participants were recruited by professionals from the educational-psychological service and alternative provisions in three Norwegian municipalities. Data were analyzed using reflexive thematic analysis.

**Results:**

The results indicate that lacking positive relationships with peers and teachers along with falling behind academically lead to school alienation and contributes to the onset or maintenance of SAPs. Similar experiences of gradual onset of SAPs, anxiety and depression, emotional and somatic distress, and conflicting feelings around avoidance were outlined. The youths’ initial experiences of support within the school setting were described as inappropriate, insufficient, or initiated too late, while attending alternative provisions appeared as a primary intervention that had enduring positive effects on attendance and engagement in academic and social activities.

**Conclusion:**

The findings highlight the need for early intervention, coherence between initiatives across support services and effective collaboration between youth, families, schools, and external services.

## 1 Introduction

Regular school attendance is associated with academic, language, and social and emotional development (1, 2). However, some students struggle with school attendance problems (SAPs), which broadly refers to difficulties in attending or remaining in school or class, as well as different types of absences (3). Research often differentiates SAPs into categories including school withdrawal (SW), school exclusion (SE), school refusal (SR), and truancy (TR). SW is generally characterized as parent-initiated absences, while SE is related to school-based decisions (4). SR and TR encompass student-initiated absences, wherein SR is often linked to internalizing and emotional difficulties (1), and TR is associated with externalizing difficulties (4). However, the symptoms of SR and TR may significantly overlap, and youth may transition between these characteristics over time (5). Thus, a broad definition of SAPs was adopted in the current study (3).

The development of SAPs is influenced by various individual and contextual factors, including emotional difficulties and symptoms of anxiety and depression (5–7), neurodevelopmental disorders (8), poor school climate (9, 10), negative relationships with teachers (5, 11), and difficulties in peer relationships and bullying (5, 11–14).

In recent years, research has increasingly focused on the role of contextual factors contributing to SAPs, and several frameworks which draw on ecological theory have been proposed to understand how SAPs emerge and develop [(e.g., (2, 15)]. One notably framework by Havik and Ingul (16) combines a systemic integrated cognitive approach, which theorizes the interplay between individual and environmental factors on youths' development (17), and the school alienation (SAL) theory (18) to understand the emergence and establishment of SR. This framework may also be relevant for the development of other types of SAPs.

According to the SAL theory, students may be alienated from three interrelated aspects of school: the learning domain, the peer domain, and the teacher domain (18). Alienation can occur in one or in all three domains, resulting in negative cognitive and affective attitudes toward schooling. Alienation from learning relates to the academic aspect of school and may involve experiencing a lack of interest or boredom during learning (19). Havik and Ingul (16) suggest that this domain is less relevant to emerging SR, as these students often enjoy and master learning. However, alienation from learning may be relevant to other types of emerging SAPs, such as TR, which is typically associated with boredom or disengagement in learning activities [(e.g., (20, 21)]. The peer domain relates to the social aspect of school and involves peer relationships. Alienation from peers is associated with feelings of loneliness, isolation, and withdrawal from classmates (19). This domain is significant, as research indicates that students with SAPs often struggle in peer- and friendship relationships (13, 14, 22). Lastly, the teacher domain encompasses both social and academic aspects of schooling. The social aspect involves supportive or non-supportive teacher-student relationships, while the academic aspect is related to instructional modes in teaching activities (19). The lack of emotional and academic support from teachers has been identified as a contributing factor to SAPs (13, 14, 22). A gradual development of SAPs may align with a SAL process, potentially reinforcing SAPs. This, in turn, could establish a vicious cycle of school avoidance, ultimately leading to prolonged absenteeism (16, 23).

In previous years, research on interventions for youth with SAPs has primarily centered on clinical approaches based on cognitive behavioral therapy (CBT), thus often delivered by services external to the school [(e.g., (24)]. However, it is recognized that SAPs may emerge from an interplay between various factors related to the youth, family, or school, and early identification and intervention is crucial to avoid problems from exacerbating (4, 25). A multidimensional multi-tiered systems of support (MD-MTSS) model has been proposed in the field of school attendance and absenteeism (26). The MD-MTSS model is based on a preventive whole-school approach and includes interventions at the universal, targeted, and intensive levels while considering the various aspects of SAPs and functioning, thus aiming to avoid what Kearney and Graczyk (26) refer to as a “wait to fail approach”.

Teachers and school staff play a key role in recognizing changes in attendance, making them essential in early identification of SAPs (27). However, a recent systematic scoping review highlighted the current lack of research focusing on school-based approaches that can be delivered and adapted by school staff themselves, instead of external professionals (28). Moreover, in their meta-analysis, Eklund et al. (29) found small effects for all attendance interventions, suggesting that existing practices are ineffective. In addition, a practitioners' review of the literature in the area of SR indicated little progress in knowledge attainment over the past decade that could assist practitioners, despite the increase in conducted studies during this time (1). This suggests a further need for studies to provide evidence to support responses to SAPs (1, 29).

Youth perspectives and inclusion in decisions that affect them are stated in Article 12 of the United Nations Convention on the Rights of the Child (UNCRC). This principle is essential for developing a full understanding of SAPs, applying to both practice and research. Existing research on youth perspectives includes a recent meta-ethnographic review from the UK (30) and a systematic review from New Zealand (31), along with additional studies from Europe and the US. Most of these studies have concentrated on risk factors related to SAPs, identifying factors related to individual, relational, and contextual domains. Individual factors included low emotional well-being, sleep difficulties, and anxiety (13, 30, 32). Regarding relational factors, experiences of poor teacher-student relationships, inconsistent adult support, poor peer relationships, peer detachment and bullying were prevalent (12, 21, 30, 31, 33–35). Among contextual factors, negative school environments, unsafe and unwelcoming classrooms, teaching quality, lack of engagement, and poor school response were emphasized as contributive (21, 31, 33, 34, 36). In addition to risk factors, some studies have identified protective factors to encourage school attendance, including a positive school climate, positive teacher-student relationships, positive peer relationships, and tailored support (20, 21, 35, 37–39).

Previous qualitative studies conducted on youth's perspectives offer important insight into the underlying dynamics of the various types of SAPs. However, the majority of existing studies center around youths' perspectives on contributive factors to SAPs while overlooking experiences of received support that could inform practitioners about effective intervention strategies. In addition, further research is required to identify commonalities among youths' experiences.

While three of the studies referenced above originate from Sweden (21, 35, 38), research that focus solely on youth perspectives on SAPs in compulsory education remains limited across the Nordic region (40). This gap is important to address as the Nordic Education Model is based on the concept of “Education for All”, emphasizing free and universal access to education, equity, equal opportunities, inclusion, and adapted learning as fundamental goals of schooling, thus differing from educational systems across other regions (41–43).

To address this, the current study conducted individual interviews with Norwegian youth who previously struggled with SAPs. The study was guided by the following research questions: (1) What reasons do youth who previously struggled with SAPs attribute to their development and persistence, and how do they interpret their experiences? And (2) How do youth who previously struggled with SAPs perceive the support they received from professionals?

## 2 Methods

### 2.1 Participants

This study used a convenience sampling strategy. Youths were recruited by professionals from the educational-psychological service (EPS) and alternative provisions in three Norwegian municipalities. In Norway, alternative provisions refer to educational settings outside mainstream schools for students requiring adapted learning environments (e.g., outdoor schools, farms, workshops). Access to these arenas is based on an individual assessment from EPS and a formal decision by the municipality in accordance with the Education Act (44), §§11-6, 11-7). Professionals in these services were selected for their ability to locate participants appropriate for the study.

To be eligible for participation, the youths had to have experienced SAPs during compulsory education (age 6–16 in Norway) to a degree that resulted in absences severe enough to necessitate external support beyond the schools' efforts. Participants were required to be over 12 years of age and to have completed primary school. Additionally, they needed to be capable of reflecting retrospectively on their experiences, either because they had overcome their challenges or because enough time had passed since compulsory education. The retrospective approach was chosen to collect youths' reflective insights into difficult past experiences with SAPs and the support they received, offering a deeper understanding of how these challenges were navigated over time.

The sample included 10 adolescents and young adults, comprising 5 girls, 4 boys, and 1 non-binary individual, aged between 12 and 22 years. All youths had experienced SAPs to a degree that included periods of complete school absenteeism, lasting between 2 and 7 years (Table 1). This diversity emerged naturally from the inclusion criteria.

**Table 1:** Participant characteristics.

Youth	Age	Duration of SAPs
Y1	12	2 years
Y2	19	3 years
Y3	17	2 years
Y4	16	2 years
Y5	19	3 years
Y6	19	4 years
Y7	17	5 years
Y8	18	2 years
Y9	22	7 years
Y10	18	2 years

### 2.2 Data collection

Semi-structured interviews were carried out individually with each youth by the first author. An interview guide with open-ended questions was collaboratively developed by all authors based on previous research. Prior to data collection, the interview guide was pilot tested with a youth in the target group (45), confirming the original guide while leading to the addition of a few follow-up questions for clarity and consistency. The interview guide addressed the following themes: school experiences leading up to struggles with school attendance, experiences of absenteeism, experiences and reflections on the support received from professionals, current situations, and hopes for the future. To mitigate interviewer bias, the guide provided a consistent foundation across all interviews, ensuring key topics were covered while allowing flexibility to explore participants' unique experiences. Interviews took place at different locations based on the participants' preferences, with one conducted digitally. The duration of interviews varied from 39 to 74 min. All interviews were recorded and transcribed verbatim.

### 2.3 Data analysis

A reflexive thematic analysis was conducted, following the stepwise procedure by Braun and Clarke (46). The six steps include (1) familiarization with the data; (2) generating initial codes; (3) searching for themes; (4) reviewing themes; (5) defining and naming themes, and (6) producing the report. NVivo 14 was used to store, code, and categorize data. The process of generating codes and themes was guided by an abductive approach, enabling the flexibility to shift between an inductive and deductive stance (47).

The analysis was conducted by the first and second author, both of whom hold prior knowledge and experience with SAPs through research. While these backgrounds offered useful insights, they also introduced a risk of influencing the interpretation of participants' narratives, particularly in the identification of themes. Hence, reflective practices were required to mitigate biases from pre-existing assumptions (48). Initially, both researchers familiarized themselves with the transcribed interviews. Then, initial codes based on sentences and terms from the data were generated. These codes were combined and sorted to develop subthemes and overarching themes relating to the research questions. Regular meetings were held between the two authors to discuss their interpretations of the data. While no major disagreements emerged, occasional differences in emphasis were resolved by revisiting the data and collaboratively refining interpretations. To further enhance reliability, the third author reviewed the themes and subthemes in light of the data material. Any uncertainties were discussed collaboratively until agreement was reached between all authors. In addition, member checks were performed by requesting feedback from each participant on the analysis results. Half of the participants responded and confirmed the study's findings.

### 2.4 Ethical considerations

The study was assessed by the Norwegian Agency for Shared Services in Education and Research (Sikt). Participants were contacted by professionals in the EPS or alternative provisions and asked if they wished to take part in the study. In line with national regulations, participants aged 16 and above received a consent form tailored to their age, containing written information about the study and the entailments of participation. For participants under age 16, parental consent was obtained through a separate consent form. Prior to each interview, the written information was repeated and reviewed orally with participants to ensure understanding and address any questions. Confidentiality was maintained throughout the research process. This was ensured by using pseudonyms and presenting demographic details in aggregate form, without linking gender to individual participants, particularly given the small sample size. All participants were informed of their right to withdraw from the study at any time.

## 3 Results

**Figure 1:**
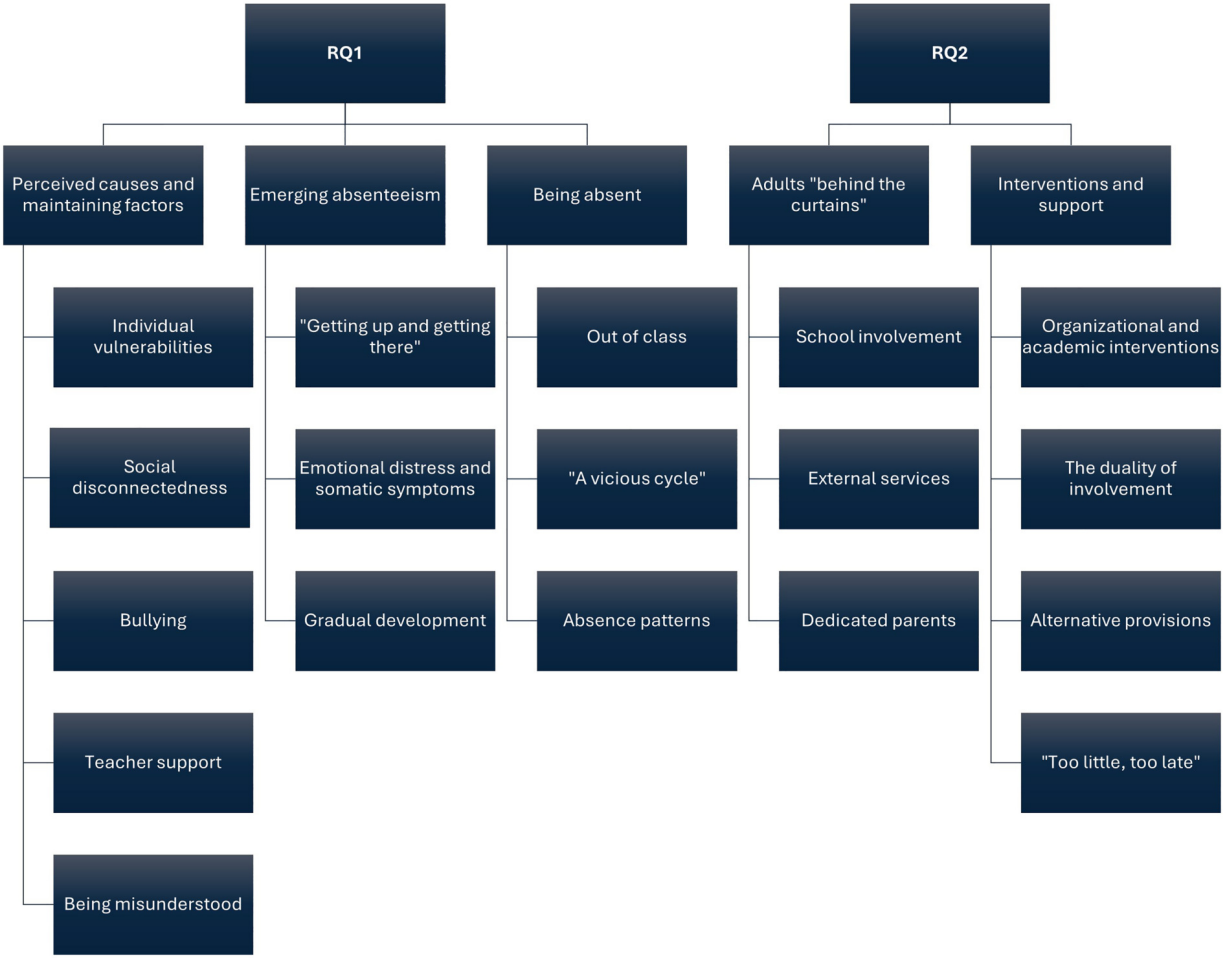
Main findings.

The analysis produced five main themes along with respective sub-themes. The first three main themes; (1) Perceived causes and maintaining factors, (2) Emerging absenteeism, and (3) Being absent were associated with the first research question, focusing on youths' perceptions of causes and experiences regarding SAPs. The remaining two main themes; (4) Adults “behind the curtains” and (5) Interventions and support were associated with the second research question, which explored youths' perceptions of support for SAPs from professionals. Figure 1 illustrates the main findings, including main- and subthemes, with detailed descriptions provided for the subthemes. Quotations, translated from Norwegian, are included to illustrate the themes and subthemes, with participating youths denoted as Y1, Y2, Y3, etc., followed by their respective age.

### 3.1 RQ1: What reasons do youth who previously struggled with SAPs attribute to their development and persistence, and how do they interpret their experiences?

This dimension consists of the three main themes “perceived causes and maintaining factors”, “emerging absenteeism”, and “being absent”.

#### 3.1.1 Perceived causes and maintaining factors

The youths shared similar perceptions regarding the reasons for their SAPs, describing a variety of factors primarily related to the school environment and individual vulnerabilities. This theme encompasses both causes described as onset and maintaining factors, which appeared to highly overlap. Subthemes include individual vulnerabilities, social disconnectedness, bullying, teacher support, and being misunderstood.

##### 3.1.1.1 Individual vulnerabilities

All youths exhibited anxiety related to school attendance, stating it as one of the primary factors contributing to their SAPs. Hence, nearly all youths had a diagnosis of anxiety (8), and half of them were also diagnosed with depression (4). Some described experiencing anxiety and depression since early childhood, even before starting school, while for others, the onset of anxiety and depression coincided with the beginning of their absenteeism.

However, in all cases, anxiety was or eventually became related to aspects of school attendance and often intensified by consequences of being absent from school. Several youths reported that their anxiety was linked to social dynamics, leading them to worry about how their peers perceived them or to interpret social interactions negatively.

“Social anxiety is like.. I was afraid of people, basically. And there are a lot of people at school” (Y4, 16).

“It was of course, anxiety, linked to like, what others thought when you were gone and stuff. When you first come back, will you get questions about it, and I didn’t have any reason, or like, any good reason, to why I was gone, so, I had to try and find excuses for that too” (Y5, 19).

Neurodevelopmental disorders such as ADHD (4), OCD (2) and ASD (1) were present with seven of the youths who all believed that features of these disorders played a significant role in their struggles to attend school. However, most of them were diagnosed a while after the onset of their SAPs, of which all expressed the belief that an earlier diagnosis would have led to necessary support from the school sooner.

“It would have made a big difference for me [.] Because I couldn’t manage socially, I couldn’t manage academically, I couldn’t succeed in any areas. And that made me think I was the problem” (Y8, 18).

##### 3.1.1.2 Social disconnectedness

Being socially disconnected emerged as a major cause for the youths' SAPs, with all of them experiencing a sense of disconnect from peers and classmates. While for some, the feeling of not belonging existed before the onset of SAP, for others, it developed as a result of being absent, contributing to the persistence of their SAPs. The youths described difficulties in accessing, making, and maintaining friendships, as well as feelings of being “outside”, “excluded” or “invisible” in peer relationships.

“I never made it in, so I didn’t really have any friends or anything social at secondary school. And that doesn’t exactly help [.] when there isn’t anything or anyone to come to” (Y4, 16).

“[.] Over time it was, in fact, loneliness. Because I started to feel like I was on the outside, of the class, because I wasn’t there as much anymore” (Y3, 17).

##### 3.1.1.3 Bullying

Seven of the youths reported bullying as a significant contributor in the development and persistence of their SAPs. The youths experienced a combination of different types of bullying (e.g., physical, verbal, relational, biased-based, and digital) that were ongoing at the time their absences occurred, and which frequently went undetected or was inadequately addressed by the school.

“I hadn’t told the teachers about the bullying, but it was visible enough, because I was probably the school’s most bullied kid at the time. And since I didn’t say anything, the teachers didn’t care” (Y2, 19).

##### 3.1.1.4 Teacher support

Most of the youths emphasized the importance of receiving support from teachers or other school staff. For instance, several of the youths' experiences with frequent teacher changes, which introduced unpredictability and contributed to the development of SAPs, underscored their need for consistent teacher support.

“In sixth grade, we got new teachers. Like all of them were new. And that came on top of everything else, so then it just all fell apart, really [.]” (Y4, 16).

When the youths were asked about their relationships with adults at school prior to and leading up to being absent, most of them brought up experiencing a lack of appropriate support. For some, this was mainly related to academic aspects, although emotional and academic support were commonly described as intertwined.

“[.] I had a hard time [emotionally], and the way the teachers handled it was to put me in a group with the ones that were academically weaker, which was certainly not what the problem was for me” (Y10, 18).

Some youths described a lack of emotional support relating to not feeling valued or cared for by teachers or school staff. This contributed both to the development of their SAPs and served as a maintaining factor for their absences.

“[.] When the adults – the principal, the office lady, the school nurse, the teachers – when everyone stopped asking me how I’m doing [.] That doesn’t exactly make you feel welcome. So, I felt very unwelcome… like, I wasn’t worthy. I felt like, excluded, from school, from the adults, from everyone” (Y7, 17).

##### 3.1.1.5 Being misunderstood

When asked about how they were met by the adults at school before their SAPs, most youths described feeling misunderstood in the sense of being underestimated, viewed differently, or perceived academically weaker than their peers.

“I wasn’t seen for who I am and, I didn’t get any more help than just getting praised for doing extremely simple things, that anyone could have done” (Y2, 19).

In continuation of these experiences, half of the youths felt defined by their challenges and experienced that their teachers perceived them as a “problem-child” or “trouble-kid”, focusing primarily on their difficulties rather than acknowledging their strengths or individuality.

“I was just looked at as like a trouble-kid. Kind of like, one who doesn’t care about school, so, they were more frustrated than worried about me” (Y8, 18).

#### 3.1.2 Emerging absenteeism

This theme was produced from descriptions of the youths' experiences regarding the emergence and the gradual establishment of SAPs and subsequent absenteeism. It consists of the subthemes “getting up and getting there”, “emotional distress and somatic symptoms”, and “gradual development”.

##### 3.1.2.1 “Getting up and getting there”

In addition to causes related to the school environment, several youths highlighted that physical barriers added to the challenge of attending school. This included getting up and out of bed in the morning or enduring long or stressful journeys to school, which increased stress levels and often led to tardiness or absenteeism. Moreover, several youths emphasized the challenge of the “doorstep test”, explaining that “the thought of school” appeared more stressful than physically being at school, with “getting there” as the most challenging aspect.

“It’s actually ok to be there, it’s better than staying home feeling guilty over not being there, right. But, getting up and getting there is the most difficult thing” (Y5, 19).

Different tactics were employed to avoid the stress associated with attending school. For some of the youths, absenteeism started with pretending to be ill to convince their parents to let them stay home.

“[.] I was a lot of fake sick. I said “oh, my stomach hurts, my throat hurts”, right, and my mom, she struggled because she was thinking, “should I respond to her emotions or should I force her to go to school” (Y6, 19).

Others took a different approach, hiding their absence by leaving the home, pretending to attend school but instead spending their time elsewhere.

“[.] I got up and left home, I just never went to school” (Y9, 22).

##### 3.1.2.2 Emotional distress and somatic symptoms

The youths described experiencing a range of negative emotions linked to their SAPs. These were frequently anxiety and stress associated with the thought of attending school, as well as sadness and guilt tied to their inability to maintain regular school attendance.

“Everything was generally very negative at that time [.] I just wasn't happy at all” (Y2, 19).

Some youths expressed frustration or anger, directed either towards their teachers, their peers or the school environment for not being accommodating, or towards themselves for struggling. Many of them had negative thoughts about themselves and described feeling “stupid”, “unworthy” and “alone”, suggesting a negative self-image and low sense of self-efficacy.

“I overthink that “you can’t do this, you are not good enough”, and stuff like that” (Y7, 17).

The negative emotions were frequently accompanied by somatic symptoms, such as discomfort, high heart rate, breathing difficulties, stomachache, nausea, or headache. These symptoms would often appear right before attending school but would diminish when school was avoided.

“Eventually it became terrible being at school, and that’s when I started getting nauseas, headaches and stomachaches, and I was barely able to breathe” (Y1, 12).

“I couldn’t get up from bed, and if I managed to get up from bed, then I could barely look at the door before feeling sick [.] And I would get frustrated with myself for not being able to go, but at the same time it was like, if I go out that door, I’m going to throw up in the backyard” (Y7, 17).

##### 3.1.2.3 Gradual development

For all youths, SAPs developed gradually in severity, with initial difficulties attending school increasing into more serious and persistent absences. A few of the youths reported having positive school experiences prior to the onset of their absenteeism, particularly during their primary school years. However, most of the youths described having a difficult time at school from the very beginning of their educational journey, which eventually led to SAPs and absenteeism. In the initial stages, SAPs typically began with difficulties attending school, gradually leading to occasional absences and eventually escalating into increased levels of absenteeism.

**“**[.] I was away once a week, then two times a week, until eventually I nearly wasn’t at school at all” (Y6, 19)

“After a while I started skipping more and more classes, and I used to, maybe come to school and sit in class and after a while I would go and sit in the locker room or something. And then, gradually, it got worse and worse, and I was less and less at school” (Y4, 16)

Except for two, all youths reported an increase in their difficulties during the transition to lower secondary school, facing larger and more unpredictable environments, as well as changes related to academic and social demands. Some of them expressed an increase in difficulties with social interactions, feeling uncertain about their friendships and struggling to fit in with peers, while others pointed to challenges in adapting to new teachers and the introduction of grades. For some, the reduced monitoring from teachers made it easier to avoid staying in class.

“In primary school, they follow up with you in another way [.] But in lower secondary school, you become responsible for getting to classes, watching the time and all that. And so, it’s much easier to sneak out and hide” (Y8, 18).

Eventually, as they continued struggling with increasing SAPs, most of the youths described reaching a breaking point, which led to more established absenteeism.

“After a while, it became really bad. I couldn’t fake through it anymore. Like, I wasn’t able to come anymore” (Y2, 19).

“I just couldn’t do it anymore. I was so broken down and burnt out and just in a really black hole” (Y10, 18).

#### 3.1.3 Being absent

This theme concerns the youths' descriptions of their experiences and the characteristics of their absenteeism, including the subthemes “out of class”, “a vicious cycle”, and “absence patterns”.

##### 3.1.3.1 Out of class

The youths' whereabouts outside of class varied. However, most of them stayed at home with their parents' knowledge. They described spending their time sleeping, playing online video games, using social media, reading books, or watching TV.

“Sometimes I just sat and played [videogames], or sometimes I slept, just to avoid it all” (Y5, 19).

Three of the youths tried to hide their absenteeism from their parents by either attending school but not showing up in class or by pretending to go to school but going elsewhere instead.

“I used to go to school because my parents wanted me to, and then I would leave school and just hang around somewhere else” (Y4, 16).

“I ended up sitting in the school toilets for many hours [.] Because if I went home and played truant, I would get disciplined for that” (Y8, 18).

##### 3.1.3.2 “A vicious cycle”

Based on the youths' descriptions, it appears that being absent from school formed a vicious cycle of conflicting feelings that was difficult to break. While avoiding school gave them a temporary sense of relief, the consequences of their absence were often followed by negative emotions and guilt.

“When I woke up and thought “I have to go to school”, it was that instant relief when I got to stay home. And then came the anxiety of not being at school again [.] So, it was a bit like, a roller coaster every day, really, of emotions” (Y6, 19).

“It’s a vicious cycle, so to speak [.] Because, when you stay home, you know you really should have been at school, so it doesn’t help to stay home either. You’re just avoiding” (Y5, 19).

Several youths emphasized that the consequences of absenteeism, such as missing school activities, falling behind academically, or developing a habit of being away from school, often led to more absenteeism, making it harder to break the vicious cycle. Hence, to cope with the negative emotions, some described a need to do something productive, such as schoolwork, while being home from school.

“[.] Because, if I did nothing, I felt more guilty” (Y3, 17).

##### 3.1.3.3 Absence patterns

Most of the youths experienced periods of increased difficulty. These experiences could be related to attending specific classes, subjects or dealing with specific teachers that the youths found uncomfortable. More commonly, however, were the challenges of returning to school after illness, weekends, or extended holidays.

“After a holiday or a weekend, I would barely manage to go to school because I had gotten used to staying home” (Y7, 17)

There were also periods or situations when the youths found it easier to attend school, frequently related to classes or subjects they enjoyed.

“When there were subjects that I liked, I always tried to attend them, specifically. Because then I managed to push myself through it more often” (Y2, 19).

### 3.2 RQ2: How do youth who previously struggled with SAPs perceive the support they received from professionals?

This dimension consists of the two main themes “adults behind the curtains” and “interventions and support”.

#### 3.2.1 Adults “behind the curtains”

This theme contains descriptions of the school staff, parents, and professionals from other support services that became involved in providing support to the youths after absenteeism had started. These professionals were commonly described by the youths as those who worked “in the background” or “behind the curtains”. Subthemes are “school involvement”, “external services”, and “dedicated parents”.

##### 3.2.1.1 School involvement

The youths were asked about the time at which their school began to worry or take action to address their absenteeism. While all youths acknowledged the schools' attempts to support them, most reported that the schools' actions were either inappropriate or initiated too late, at a point when their absenteeism was already well-established.

“They didn’t initiate anything before it had gotten extreme” (Y2, 19).

Several youths described feeling that despite the schools' efforts to help and support them, they encountered a lack of competency and understanding from the staff regarding their difficulties. This frequently led to measures feeling insufficient or not tailored to their individual needs.

“There were a lot of people who tried to help, but I think many of them didn’t have enough competency to actually be able to help me” (Y8, 18).

“[.] They involved themselves in the wrong way, so to speak. They didn’t understand the problem” (Y10, 18).

##### 3.2.1.2 External services

As school initiatives proved insufficient, all the youths eventually received additional support from external professionals, including those working in educational, psychological, or social services within the community. However, it appeared that the youths were not always in direct contact with these professionals.

“I think as a child you don’t know all that is being done behind the curtains” (Y10, 18).

A few of the youths described experiencing inconsistency in the professionals they encountered, with frequently changing personnel making it difficult for them to build trust and feel supported.

“There were nine, ten different people that came in and out all the time, and who were talking to me [.] I saw each of them once and then they just disappeared” (Y1, 12).

In addition, all youths received assessment and support from the child and adolescent mental health service (CAMHS) and had been in direct contact with a therapist. However, the youths frequently described a lack of coherence between the support provided by CAMHS and what they received from their school, with therapists prioritizing emotional well-being and schools mainly focusing on attendance and academics. A few of the youths expressed the belief that the school staff lacked awareness of their mental health needs and how to accommodate them appropriately within the school environment, resulting in fragmented support.

“I always had a therapist, so, I think they thought, “we don’t need to bring these issues at school further up the system because you have a therapist” (Y8, 18).

##### 3.2.1.3 Dedicated parents

Several youths expressed having supportive and actively involved parents who initiated contact with the school and took on a coordinating role in the collaboration with both the school and other support services. Some youths recalled instances of tension or conflict between their parents and the school, often due to their parents' perception that the school was not providing sufficient support.

“I didn’t know it at the time, but there was a constant friction between my parents and the school, on that topic” (Y9, 22).

Consequently, half of the youths reported that their parents attempted at least one school change to improve their situation. For one of them, transferring to another school significantly improved their experience. However, most noted that this did not prove to be a lasting solution.

“I changed schools [.] and I had it good for two years. But then it fell apart again” (Y6, 19).

#### 3.2.2 Interventions and support

This theme was generated from the youths’ descriptions of the types of interventions that were arranged by school staff and other professionals to support them and consists of the subthemes “organizational and academic interventions”, “the duality of involvement”, “alternative provisions”, and “too little, too late”.

##### 3.2.2.1 Organizational and academic interventions

Youths described a variety of organizational and academic interventions initiated at the school. Academic measures included adjusting academic demands and arranging home-based schoolwork. Organizational measures involved adjustment of school hours (e.g., starting school later or leaving school earlier), smaller group work, reviewing the weekly schedule in advance to increase predictability, and mobilizing an adult at school for assistance. Noteworthy, mobilizing an adult was perceived as the most helpful initiative, but this was largely dependent on whether the youths had a good relationship with the adult.

“There was one woman, I think she worked for the municipality or something, and the school had talked to her [.] She came and met me, at home, and followed me to school. And I wasn’t a fan of it at all [.] And I remember some days when she couldn’t come, my contact teacher did it instead of her. But, when he came, it was ok, because we were buddies” (Y5, 19).

The most common organizational measure reported by all except one youth was being assigned a separate room at school. Although this initially lowered the threshold for attending school, it was not helpful long term. Several youths described feeling more “alone” or “isolated” while in the separate room and noted that this increased the threshold for returning to the classroom with their peers. They also described receiving little help from teachers while in the separate room, who often did not have the time or capacity to provide individual support.

“In the beginning it was like, I was still in class, but I had access to the separate room, so like halfway through the day I could go and sit there. [.] But after a while I just came and stayed the whole day in that room” (Y3, 17).

“They said I could come to school, sit in a separate room, and stay there if it was too difficult to be in class. But when I did that, I ended up sitting there alone for three hours without anyone checking in on me” (Y6, 19).

##### 3.2.2.2 The duality of involvement

Nearly all youths described having the opportunity to engage in decision-making before interventions were implemented. However, there appeared to be a duality of this involvement. For instance, half the youths felt that they were given too much flexibility and found it difficult expressing a clear idea of what might be effective, as they felt uncertain about this themselves.

“Is there anything that could help, is there anything we can do, is there something that will help you”. I have no idea. I haven't experienced anything that helps [.] But at least they asked me. I just couldn't help them help me” (Y6, 19).

Others felt their involvement was limited to responding yes or no to already proposed interventions by the school, rather than actively being involved in discussing possible adaptations. At times, they also experienced an expectation that they should support the proposal.

“I didn’t get to decide a lot. They were like, came in, had a meeting with like, the principle, the head teacher, whoever it was, and they were like “we decided this, do you agree”, and you were pushed to say yes” (Y7, 17).

##### 3.2.2.3 Alternative provisions

For seven of the youths, the support that was offered within the school context was not sufficient to help them return to school. Eventually, these youths were offered alternative provisions, which provided them with a sense of finally receiving the help they needed.

“I remember feeling, ok, now things can change. Now there is someone other than the school nurse helping me. It felt more, real, sort of” (Y6, 19).

All of them described their time at the alternative provisions positively. Organizational aspects such as higher student-adult ratio, flexible time schedules, increased involvement and predictability, resources, and time were emphasized.

“The fact that they had a lot of time. Time to build relations and to build me back up. Because I was scattered to pieces. And they glued me back together, little by little” (Y10, 18).

The youths also described feeling validated and cared for by the staff at the alternative provisions, highlighting their knowledge and competency regarding their difficulties.

“When I talked about something, they knew, and they had heard it before” (Y6, 19).

Feeling adequately met and understood appeared to contribute to youths' motivation to attend school and engage in academic and social activities, and all of them reported that their attendance and engagement increased as a result. Moreover, the same seven youths expressed that attending an alternative provision was the primary intervention that had a positive long-term impact on them.

“That school was the only thing that helped me actually go to school, be at school, get grades and contribute in class” (Y7, 17).

“I am really thankful for that school. If it wasn’t for them, I wouldn’t be here today. Simple as that” (Y9, 22).

##### 3.2.2.4 “Too little, too late”

When reflecting on how their SAPs were addressed, most youths described deficiencies in the support they received from the school and other support services. Nearly all of them expressed that they wished that they had received support earlier and felt that this would have prevented their absenteeism from establishing.

“If there is anything I would have changed, it’s the fact that the measures should have been initiated many years earlier [.] Like I said, too little, too late” (Y9, 22).

## 4 Discussion

This study aimed to achieve an understanding of the retrospective experiences of Norwegian youth with a history of SAPs in compulsory education. The study was conducted in the context of the Nordic education system, characterized by free schooling, universal access, equity, and inclusive education policies (41, 43). These systemic features distinguish the Nordic context from other international education systems, affecting how SAPs are experienced and addressed across contexts [(e.g., (42)].

In the current study, two research questions were addressed: (1) What reasons do youth who previously struggled with SAPs attribute to their development and persistence, and how do they interpret their experiences? And (2) How do youth who previously struggled with SAPs perceive the support they received from professionals?

To investigate the first research question, youths' perceptions of the onset and maintaining factors of SAPs, as well as their interpretations of their experiences were explored. First, anxiety was reported as one of the primary reasons for their SAPs, with nearly all having diagnosis of anxiety and depression. This corresponds to earlier research linking mental health difficulties, such as anxiety and depression, to SAPs (5–7). There was also a high prevalence of neurodevelopmental disorders (ADHD, OCD, ASD) among the youths, which were commonly diagnosed after the onset of their SAPs. These late diagnosis may have led to insufficient accommodations within the school environment, and thus delayed interventions aimed at preventing the development of SAPs. Recent research has found neurodevelopmental disorders to be pervasive among youth with SAPs and suggests they might also increase the risk of developing SAPs (8, 49). In addition, youth with neurodevelopmental disorders are more likely to experience mental health difficulties, especially anxiety and depression (50). This may explain the high frequency of both neurodevelopmental disorders and mental health diagnosis in the current sample.

Another contributor to the youths' SAPs were issues related to the social aspect of school, which included experiences of various types of bullying, difficulties in friendship and peer relationships, and perceiving themselves as on the outside or as excluded from the peer group. Negative experiences with peers align with findings from previous studies involving youth with SAPs (13, 14, 33). Moreover, research consistently indicates that prolonged bullying has a range of harmful consequences related to health and psychosocial issues, with anxiety and depression being the most commonly reported mental health difficulties (51). Bullying has also been identified as a risk factor for SAPs in several studies, as it increases the risk of anxiety and avoidance (12, 21, 34). Experiences of social disconnectedness as a result of bullying and friendship difficulties may relate to the peer domain of the SAL theory (16). Students who feel alienated from their peers often experience loneliness, isolation, and withdrawal from classmates (19). Our findings suggest this sense of disconnection may worsen with increased absenteeism, which contributes to the persistence of SAPs. These findings collectively underscore the importance of school-wide proactive approaches that foster inclusivity, positive peer relationships and psychosocial safety to mitigate the risks of bullying and social disconnection.

Inadequate support and low expectations from teachers and other school staff was frequently reported by the youths, persisting as their SAPs and related difficulties emerged. Previous studies have found that both positive and negative teacher-student relationships significantly impact SAPs, serving as a protective buffer or as a risk factor [e.g., (22, 30, 31)]. In the current study, several youths reported feeling unsupported and academically underestimated, underscoring evidence from research showing that high and clear expectations from teachers are linked to lower levels of unauthorized absenteeism (9, 39). Many of the youths additionally perceived a lack of emotional support from teachers, feeling undervalued and misunderstood or labeled as a “problem”. According to Torrens Armstrong et al. (52), professionals' perceptions and labelling of students with SAPs have the potential to either exacerbate their difficulties or allow for positive support. This has important implications as it stresses the importance of professionals adopting a strength-based approach in their work to support youth with SAPs, emphasizing their potentials and interests rather than their deficits or struggles, to support the development of effective interventions. When youth perceive their teachers as academically and emotionally unsupportive, this may lead to them feeling alienated from their teachers which increases the risk of SAPs (18, 19). These findings align with previous studies suggesting that lacking positive relationships with teachers may contribute to establish and worsen absenteeism (11, 20, 21).

The youths experienced a gradual development of their SAPs, accompanied by the presence of physical barriers, negative emotions, and somatic symptoms at the thought of attending school. These characteristics align with the typical SAP development described by Kearney et al. (3). Also consistent with the experiences outlined in this study, previous studies have demonstrated the presence of somatic and emotional distress triggering avoidance in youth with SAPs (13, 32), as well as negative self-perceptions and low self-efficacy regarding school attendance (30). A lack of belief in the ability to cope with school demands and stressors may function to increase avoidance and gradually lead to established absence (16). The youths reported that their absenteeism emerged gradually and sporadically but tended to establish after reaching a breaking point where stressors exceeded their capacity to cope. In line with the findings of Baker and Bishops' (13) study, several youths experienced increased school absenteeism when transitioning from primary to lower secondary school, which introduced new academic and social challenges. To accommodate these challenges, it is important that school staff are aware of the risks youth face when transitioning to lower secondary school, such as adapting to a larger, more complex environment, navigating new social dynamics and academic demands, and coping with increased unpredictability (25).

As the youths' SAPs established, most of them eventually stayed home with their parents' knowledge, engaging in activities such as sleeping, reading, or using the internet. Similarly, Rohrig et al. (53), found that youth were commonly home alone using electronics when missing school. While the internet and social media might offer a temporary sense of connectedness, it may also have a prolonged negative effect on anxiety and social functioning in the real world, thus reinforcing absenteeism (32). This tendency relates to the youths' descriptions of conflicting feelings of temporary relief followed by guilt and anxiety from avoiding school, which also aligns with patterns described in a previous study by Dannow et al. (14). Although avoiding school provided an instant relief, anxiety towards school is likely to worsen as absenteeism extends, creating a vicious cycle of avoidance (23). Consequently, to cope with the guilt of not attending, youths often felt the need to engage in productive activities, such as schoolwork. This aligns with literature descriptions, noting that youth with SAPs often value learning and perform well academically (23), but may also experience learning difficulties, possibly due to falling behind on schoolwork because of absenteeism (15).

The vicious cycle of SAPs can be considered in relation to a SAL process (16, 19). In the present findings, alienation from peers emerged from youths' experiences of exclusion, bullying, and social disconnection. Alienation from teachers appeared in the youths' descriptions of feeling misunderstood, as well as emotionally and academically unsupported. Several youths also felt academically underestimated and perceived that teachers held low expectations of them, which along with the experience of falling behind on schoolwork, contributed to alienation from learning. Feeling alienated from these domains may lead to the development of SAPs, but continuous SAL may also reinforce absenteeism, acting as a maintenance factor for SAPs (16). This process can be illustrated by the youths' experiences with periods of increased difficulty, such as after staying home due to being ill or after a weekend or extended holiday. These instances have been identified as situations that pose a risk for developing SAPs (25). For the youth, the routine of staying home following these situations intensified the feeling of disconnection from school, making it more challenging to return. Nonetheless, youths reported that attending school was easier in subjects they found enjoyable, underscoring the importance of professionals considering whether the youths' absence and attendance has a specific pattern to ensure a strength-based approach to intervention strategies (23).

The study's second research question aimed to address youths' perceptions of the support they received from professionals. Commonly, schools initially attempted to address the youth's SAPs, but most described these actions as inappropriate or delayed. School staff are effectively positioned to identify students with emerging SAPs, but this presupposes that they are competent in recognizing early signs and risk factors (25). Delayed or inappropriate support may allow SAPs to establish and facilitate a “wait to fail approach” (26). As a consequence of delayed intervention by the schools, all youth reported that external professionals from a variety of educational, psychological, or social services were brought in to extend support, suggesting a need for targeted interventions (26). However, in contrast to their direct engagement with school staff and CAMHS therapists, the youths struggled to differentiate between other professionals and to understand who had contributed what support, as their involvement was often perceived as taking place “behind the curtains”. Still, the findings revealed inconsistencies in the support provided by external professionals, including CAMHS, particularly regarding their coordination with school initiatives. These findings contrast with recommendations from a recent systematic review highlighting that successful outcomes of school-based approaches toward SAPs were highly dependent on effective communication and cooperation between school staff, external services, and families (28). As a result of inconsistent collaboration, several youths described their parents as having to take on a coordinating role in collaborating with the school and seeking help from external support services. Dannow et al. (14) reported similar findings and emphasized that because parents do not have the sufficient qualifications to take on the primary responsibility for solving their child's difficulties, it could at worst result in them becoming a maintaining factor in their child's SAPs. However, it is probable that parents may at times be driven to take on this responsibility due to inadequate support offered by the school. This is reflected in the current study by the frequency of school changes initiated by parents as an attempt to improve their child's situation. Similarly, previous studies have indicated that youth with SAPs change schools more frequently than those without (15), and Heyne et al. (24) note that school changes may be warranted in cases where parents have lost confidence in the school.

One common school-level response to the youths' SAPs involved mobilizing an adult for assistance at the start of the school day. However, the perceived effectiveness of this was largely dependent on the youth's relationship with the adult, which underscores the significance of positive relationships between youth with SAPs and the professionals supporting them, as these relationships are crucial for effective intervention (27–29). The most frequent measure was getting assigned a separate room at school, initially perceived helpful as it increased school attendance. However, youths described becoming increasingly detached from the school environment, in which they felt more disconnected and alienated from peers and teachers. Rohrig et al. (53) suggest that alternative options, such as separate rooms, should serve as a steppingstone between staying at home and staying in the classroom. In this study, however, the use of a separate room appeared counterproductive as it sustained or potentially increased the threshold for returning to the classroom. This finding supports the broader conclusions drawn in Boaler and Bond's (28) systematic review, which noted a significant lack of empirical evidence supporting the effectiveness of interventions delivered by school staff. Hence, more research is needed to identify and validate such strategies.

For most of the youth, school-level responses proved insufficient. Instead, alternative provisions had enduring positive effects on their attendance, grades, and social engagement. These outcomes were attributed to higher student-adult ratios, flexible schedules, increased predictability, involvement, and staff competency, all of which contrasted with their experiences in mainstream school. Likewise, both Wilkins (39) and Nuttall and Woods (27) noted the effectiveness of these factors in alternative provisions for youth with SAPs. Moreover, school or class size, flexibility and predictability have been reported to impact SAPs in mainstream schools (14, 22). On one hand, variations in these factors are a part of regular school attendance, requiring students to adapt to school expectations. However, schools have a significant responsibility in adjusting demands to accommodate students' individual needs, especially those who display difficulties. Therefore, mainstream schools might benefit from adapting strategies used in alternative provisions to improve support provided to students with SAPs. For instance, ensuring students' sense of flexibility and predictability in decision-making regarding their SAPs, in line with Article 12 in United Nations Convention on the Rights of the Child (UNCRC), is essential. While all youth in the current study had the opportunity to participate in decision-making, a prominent finding was that involvement was often twofold. Some experienced excessive flexibility that led to passivity because they lacked the knowledge or confidence to make informed suggestions, while others were presented with predefined measures they were expected to accept. These contrasting experiences emphasize the need for a balanced approach to youth involvement in decision-making. This may require professionals to offer guided choices while also encouraging an open discussion to ensure the achievement of meaningful participatory involvement. According to our findings, this balance seemed more attainable within the alternative provisions, likely due to higher resources and competency among staff regarding SAPs. The competency of school staff is critical for successfully implementing interventions toward SAPs, facilitating positive student-teacher relationships (27, 28), and early detection of risk factors (25). In the current study, all youth reported that support efforts were initiated too late, contradicting the emphasis on early interventions in the literature on SAPs, and corresponding to the notion that SAPs become increasingly difficult to reverse as they are allowed to establish (26, 28, 29).

## 5 Strengths and limitations

The current study is not without limitations. First, given the qualitative design, the study has limited possibilities for generalization across context, although it may hold transferability to comparable educational environments. The findings should, however, be interpreted within the Nordic educational context, where structural features may limit the direct transferability of findings to educational systems with differing structures, resources, or inclusion policies. Moreover, recruitment of participants who previously struggled with SAPs proved challenging, as evidenced by the small sample size and the withdrawal of four youths from the study. The small sample size may have introduced self-recruitment bias, as youth who agreed to participate in the study might have other characteristics than those who declined, potentially influencing the results. However, clear criteria for participant selection, particularly regarding SAP severity, could reduce the impact of these limitations and increase the validity of the findings. Moreover, the current study relied on self-reported retrospective accounts, which could result in data being shaped by subjectivity or inaccuracies in the youths' recollections of past experiences. With self-reported data, there is also a risk that social desirability and response bias have an impact on the data. A strength of this approach, however, was that it allowed for reflective insights into the youths' first-hand experiences, offering valuable perspectives on their challenges and experiences of support, which have received limited research attention in the past.

## 6 Conclusion

This study investigated Norwegian youths’ retrospective experiences of SAPs and received support from involved professionals. First, youths outlined similar experiences of gradual onset of SAPs, accompanied by emotional and somatic distress, and conflicting feelings about school attendance. The study mainly identified individual (e.g., mental health and neurodevelopmental diagnoses) and relational (e.g., friendship difficulties, bullying, inadequate teacher support) factors as contributing to the development of SAPs. Findings indicate that relational factors, such as poor peer- and teacher relationships, along with falling behind academically, may lead to SAL which contributes to the onset and maintenance of SAPs. Second, youths' initial experiences of support within the school setting were perceived as inappropriate, insufficient, or delayed. Support from professionals from external services (e.g., educational, psychological, or social) was perceived as inconsistent and uncoordinated with school initiatives. Hence, most youths eventually attended alternative provisions, which had enduring positive effects on attendance and engagement in academic and social activities.

These findings underscore the significance of fostering early intervention efforts in schools, including protocols for systematic attendance monitoring to detect and support students at risk. Efforts should focus on strengthening positive teacher- and peer relationships and providing individualized accommodations at the school level, to mitigate the risk of SAL and SAPs among vulnerable students. Effective collaboration between the youth, family, school and external professionals, with an emphasis on coherence between support initiatives, is essential to ensure timely and adequate intervention. For instance, regular, structured meetings between different stakeholders could be implemented to develop and monitor the effect of interventions. Moreover, the findings suggest that incorporating strategies from alternative provisions, such as flexibility, predictability, and involvement, into mainstream school support efforts may enhance the initial support provided to youth experiencing SAPs. Lastly, increased resource allocation for flexible, school-based interventions and interdisciplinary collaboration teams may be an important consideration for policymakers.

The current study adds to previous studies addressing youth perspectives on SAPs, identifying contributive factors among individual, relational, and contextual domains. In addition, youths’ experiences of receiving support were investigated, offering insight into the perceived effectiveness and availability of interventions. To further explore support initiatives, future studies should include professionals' and other stakeholders' perspectives. Moreover, studies should focus on identifying and evaluating the effectiveness of intervention strategies offered by mainstream schools in cases of SAPs, as there seems to be lack of evidence-based practices provided within the school setting.

## Data Availability

The datasets generated and analyzed for this study obtain sensitive qualitative data from interviews with a vulnerable population. Due to ethical and privacy concerns, as well as the risk of participant identification, the raw data cannot be shared publicly. Access to anonymized data may be granted upon reasonable request.
